# Unconventional Protein Secretion Dependent on Two Extracellular Vesicles: Exosomes and Ectosomes

**DOI:** 10.3389/fcell.2022.877344

**Published:** 2022-06-09

**Authors:** Jacopo Meldolesi

**Affiliations:** ^1^ The San Raffaele Institute, Vita-Salute San Raffaele University, Milan, Italy; ^2^ The CNR Institute of Neuroscience at Milano-Bicocca University, Milan, Italy

**Keywords:** endocytosis, multivesicular body, exocytosis, pinching off, navigation, vesicle fusion, interconnected networks

## Abstract

In addition to conventional protein secretion, dependent on the specific cleavage of signal sequences, proteins are secreted by other processes, all together called unconventional. Among the mechanisms operative in unconventional secretion, some are based on two families of extracellular vesicle (EVs), expressed by all types of cells: the exosomes (before secretion called ILVs) and ectosomes (average diameters ∼70 and ∼250 nm). The two types of EVs have been largely characterized by extensive studies. ILVs are assembled within endocytic vacuoles by inward budding of small membrane microdomains associated to cytosolic cargos including unconventional secretory proteins. The vacuoles containing ILVs are called multivesicular bodies (MVBs). Upon their possible molecular exchange with autophagosomes, MVBs undergo two alternative forms of fusion: 1. with lysosomes, followed by large digestion of their cargo molecules; and 2. with plasma membrane (called exocytosis), followed by extracellular diffusion of exosomes. The vesicles of the other type, the ectosomes, are differently assembled. Distinct plasma membrane rafts undergo rapid outward budding accompanied by accumulation of cytosolic/secretory cargo molecules, up to their sewing and pinching off. Both types of EV, released to the extracellular fluid in their complete forms including both membrane and cargo, start navigation for various times and distances, until their fusion with target cells. Release/navigation/fusion of EVs establish continuous tridimensional networks exchanging molecules, signals and information among cells. The proteins unconventionally secreted *via* EVs are a few hundreds. Some of them are functionally relevant (examples FADD, TNF, TACE), governing physiological processes and important diseases. Such proteins, at present intensely investigated, predict future discoveries and innovative developments, relevant for basic research and clinical practice.

## 1 Discovery of Unconventional Protein Secretion

The existence of specific protein secretion, a property of all types of cells, was already known at the beginning of the last century. At that time, however, the mechanisms of the process were unknown and remained so for decades. Information started to emerge at the beginning of 1960. Digestive enzymes of pancreatic acinar cells, in the course of their synthesis by bound polyribosomes, are transported to the lumen of the endoplasmic reticulum (ER). Segregated enzymes were found to move to the Golgi complex (GC), and then concentrate in the cargo of secretory granules, which accumulate in the cytoplasm during jejunum. Upon food intake or cell stimulation the granules were found to undergo exocytosis by fusion of their membrane to the plasma membrane, followed by extracellular discharge of their cargos (Palade et al., 1962; Caro and Palade, 1964). Subsequent studies demonstrated that mechanisms analogous to those of the pancreas operate also in other cell types (Schramm, 1967; Meldolesi et al., 1978). In addition, the general processes governing the various steps of the secretory pathways started to be discovered. The first step, concerning the signal recognition particles of the ER membrane surface, was shown to induce translocation of pre-secretory proteins into the corresponding ER lumen. For years, cleavage of signal sequences (Walter and Blobel, 1981; Müller et al., 1982; Walter et al., 1984) was considered necessary for the development of a secretion now called conventional or canonical.

For over 2 decades, progress about secretion concerned only the conventional pathway. Around 1990, however, evidence incompatible with that interpretation begun to emerge. Cytosolic proteins lacking a signal sequence in their gene, such as interleukin-*β*1, bacterial enzymes and growing numbers of proteins and factors, were shown to be discharged by unconventional secretion (Rubartelli et al., 1990; Rubartelli et al., 1993; Akatsuka et al., 1995; Nickel and Rabouille, 2009). Initially these processes were proposed to activate, in the plasma membrane, various types of pore permeable to secretory proteins (Rubartelli et al., 1990; Nickel and Rabouille, 2009). Plasma membrane pores, together with a channel in the ER/Golgi membranes, are still considered of relevance in the trans-membrane transport of proteins lacking signal peptides (Rabouille, 2017; Zhang et al., 2020). At present, however, the major pathways of unconventional protein secretion appear based on the participation of various types of organelles (Rabouille, 2017; Gruenberg, 2020).

**FIGURE 1:**
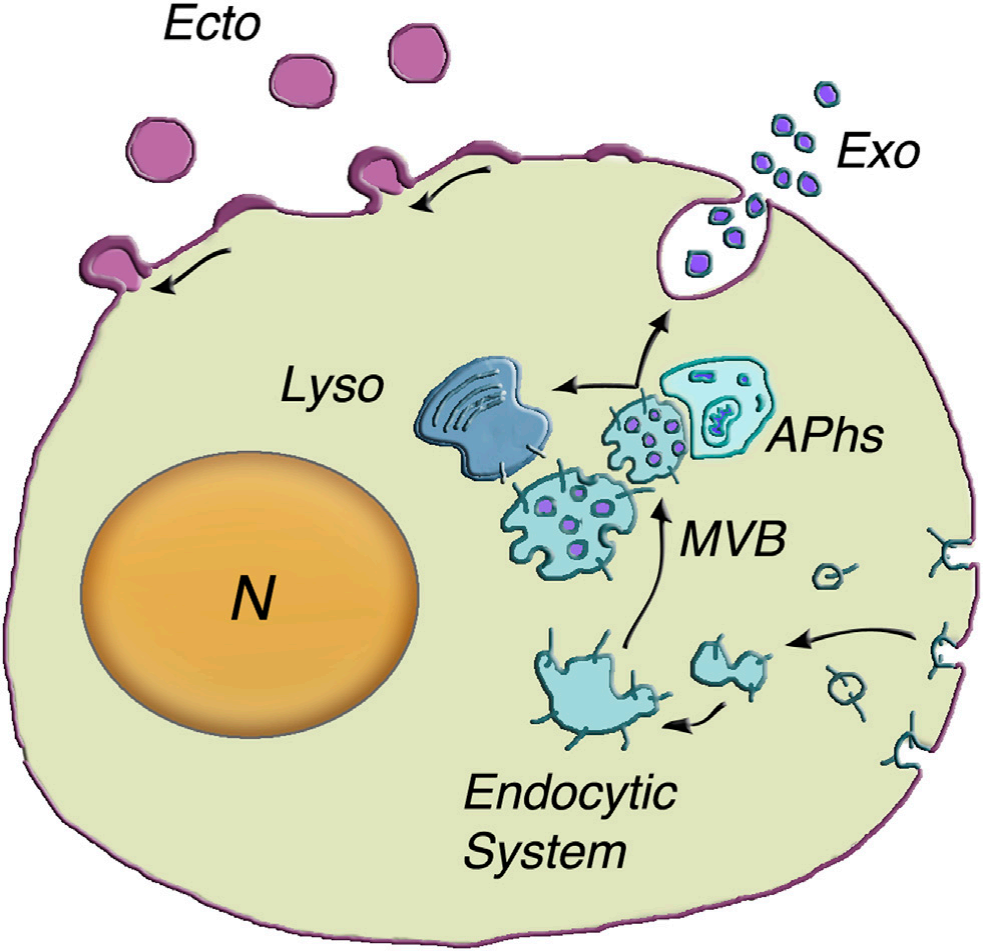
Generation and release of both exosomes and ectosomes. The dependence of exosome generation on the Endocytic System is illustrated at the center of the figure. The multivesicular body (MVB) is an endocytic vacuole occupied by vesicles corresponding to distinct intraluminal vesicles (ILVs) filled by specific cargo accumulated from the cytosol. The inward budding of the vesicles is followed by their fission and the release of ILVs into the MVB lumen (50–150 nm diameter). Upon their generation, the MVBs can proceed in two alternative directions (arrows): towards lysosomes (Lyso) or towards the plasma membrane. Their close interaction with autophagosomes) can induce reciprocal exchange of cargo components and ensuing increased fusion in response to stimulation. MVB exocytosis is followed by prompt extracellular release of vesicles, now named exosomes (Exo). The assembly and release of ectosomes, on top left of the cell surface, take place at the plasma membrane. The initial step is the assembly of membrane larger microdomains (100–400 nm in ectosome diameter). Their composition is distinct from that of the plasma membrane of origin and partially similar to that of ILV membranes. Concomitantly specific cargos, composed of proteins, lipids and nucleic acids, accumulate in the vesicle lumen. Upon their rapid outward curvature and budding, the ectosome vesicles (Ecto) undergo pinching off and shedding to the extracellular space. N = nucleus. Modification of the image reproduced with permission from Meldolesi (2018).

In the cytoplasm, at least three types of organelles, involved also in other important functions, are known to participate in unconventional secretion. These organelles include: lysosomes, with many enzymes necessary for catabolism; autophagosomes, that in their journey from ER to lysosomes fuse with vesicles and integrate cytosolic molecules and nutrients (Zhao and Zhang, 2019); and multivesicular bodies (MVBs), the only endocytic vacuoles known to accumulate large numbers of small intraluminal vesicles, the ILVs (Karim et al., 2018) (Figure 1). This review is focused on two types of extracellular vesicles (EVs), expressed by all types of cells and active in unconventional secretion: ILVs (called exosomes upon their release to the extracellular space) and the larger ectosomes (also known as microvesicles and microparticles). The exosomes are released upon exocytosis of MVBs; the ectosomes, independent of MVBs, are generated and released by shedding from the plasma membrane.

## 2 Presentation of ILV/Exosomes and Ectosomes

Before the generation and function of ILV/exosomes and ectosomes, illustrated in the next two Sections, the vesicles are presented in their general properties. Let’s start with MVBs and ILVs. Upon its ILV accumulation, MVBs undergo their maturation. Their destiny is two fold. Upon interaction with the Rab7 ortolog Ypt2 and the multisubunit tethering complex HOPS, a fraction of MVBs proceed to specific fusion with lysosomes by a process including the Qa-SNARE Pep12. The ILVs discharged to the lysosomal lumen are thus exposed to hydrolases for catabolism (Karim et al., 2018). Other, apparently distinct MVBs, by interacting with actin and microtubule cytoskeleton move towards the microtubule-organizing center and then to the plasma membrane. From such location they undergo exocytosis in response to appropriate cell stimulation (Hessvik and Llorente, 2018; Raudenska et al., 2021). Details about their discharge are illustrated in the following Section 3.1. New aspects of the processes involving MVBs include a co-operation with autophagosomes (Figure 1). Comparative studies of exocytosis with and without autophagy inhibitors have revealed that, during their intracellular traffic, MVBs and autophagosomes interact with each other with exchange of their cargo proteins. When autophagosomes are not available, exocytosis of MVBs is greatly reduced (Bebelman et al., 2020). The interaction of the two organelles plays therefore an integrated form of unconventional secretion.

Stimulation of the MVB exocytosis discussed so far is followed by the diffusion of many ILV/exosome vesicles, from the lumen to the extracellular space (Figure 1) (Rabouille, 2017; Bebelman et al., 2020; Gruenberg, 2020; Zhang et al., 2020; Gurung et al., 2021; Ras-Carmona et al., 2021; Raudenska et al., 2021). In many cases such process is accompanied by the release of ectosomes, the other type of EVs, generated not within the cytoplasm but at the plasma membrane. Being a single process, ectosome generation and release are not presented here. They will be illustrated in the subsequent Section 3.2, i.e. just after 3.1, the Section dedicated to the nature and generation of exosomes.

In order to complete the presentation of the two EV types, I intend to emphasize three properties of their research. For many years, simple procedures such as ultracentrifugation, precipitation, filtration, chromatography, and immune-affinity-based approaches, have been employed to separate the two types of vesicle present in extracellular fluids. However, these approaches yielded poor results (Shtam et al., 2020). As a consequence, research interests were primarily focused on only one type, the exosomes. Results about ectosomes remained marginal. Among reported differences only one was conclusive, concerning the different size of the two types of vesicles, with diameters between 30 and 150 nm for exosomes, between 100 and 400 (or more) nm for ectosomes. Other differential properties, including protein and RNA composition, fusion to cell targets, and functional effects, became convincing only upon development of better isolation procedures (see for example Meldolesi, 2018; Van Niel et al., 2018; Royo et al., 2020). The results obtained by such procedures have shown the two types of vesicles to differ considerably in some cells, and not so much in other cells (Pizzirani et al., 2007; Kowal et al., 2016; Meldolesi, 2018; Van Niel et al., 2018; Cocozza et al., 2020; Royo et al., 2020; Lim et al., 2021; Mathieu et al., 2021).

At variance with granules and vesicles of conventional secretion, of which only cargos are discharged upon cell activation, the discharge to the extracellular space occurs by whole EVs, composed by cargos bound by their membrane. In fact, membranes are essential for the EV navigation and for its specific binding and fusion to their target cells. As long as the EVs are intact their cargo components: many proteins, various types of RNAs, short DNA sequences, a few types of lipids, metabolic molecules and various ions, remain largely assembled.

Exosomes and ectosomes are unconventional secretory vesicles, released by all types of cells. Their heterogeneity depends on their distinct differentiation and also on their cells of origin. At variance with the other cellular organelles, discharged exosomes and ectosomes are very resistant, they withstand harsh conditions such as those of the human stomach. Based on these properties, their origin has been hypothesized more ancient than that of intracellular organelles (Askenase, 2021). For these unique natural properties, and also for their engineered treatments of biotechnological relevance, EVs are considered of interest for a number of pathologies, including cancers, neurodegenerative and viral diseases. These properties deal not only with unconventional secretion, but also with other functions that will not be presented here. Nevertheless, they are of high medical relevance, already presented by a vast literature.

## 3 Development of Secretory Vesicles Within the Cell

Before detailed illustration of EVs, let’s consider the nomenclatures valid for the two secretory vesicles, ILVs and ectosomes within the cell, exosomes and ectosomes, upon their release. Relevant studies have emphasized the heterogeneity of single EV populations (Pizzirani et al., 2007). In the future, therefore, unexpected results dependent on the coexistence of vesicle subtypes cannot be excluded. In various articles the EVs, in addition to exosomes and etosomes, have been reported to include larger structures, with diameters of one um and more. Most of these structures, however, are membrane fragments and cell debris. Because of their non-vesicular nature they will not be considered in the present review.

The initial life of the two EVs is profoundly different. They are not only generated at different sites and by different processes (Figure 1), they differ also in their subsequent intracellular life: long lasting for ILVs segregated within MVBs; very short for ectosomes. In view of these differences, the intracellular life of the two vesicles, focused on the mechanisms of their occurrence, are presented separately.

### 3.1 ILVs/Exosomes

As already mentioned in Section 1, generation of ILVs induces the conversion of normal endosomal vacuoles into peculiar MVBs. The conversion starts with two processes: budding inside the vacuoles of the small membrane protrusions concomitantly loaded with their cargo content.

**FIGURE 2:**
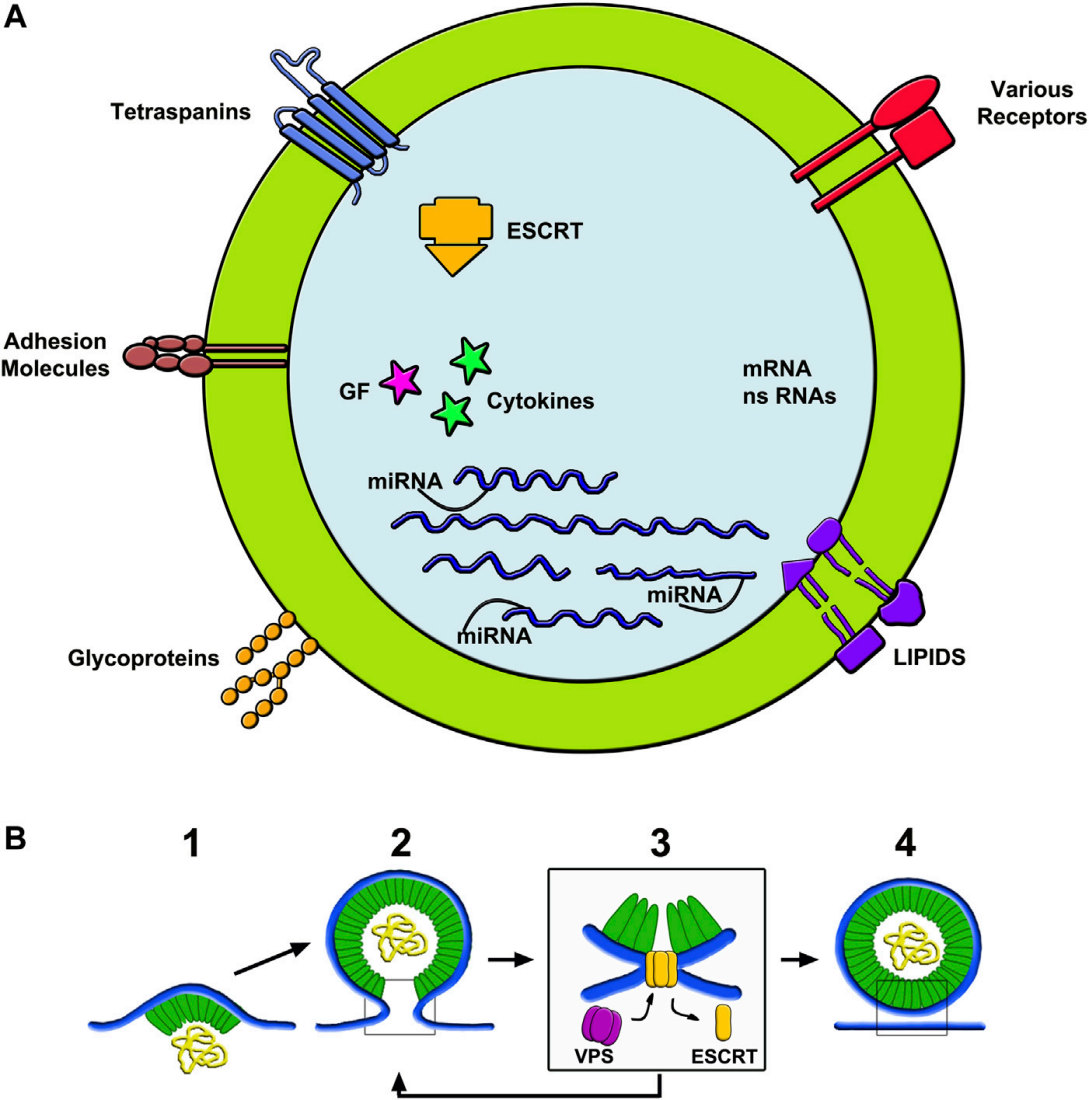
Structure of exosomes **(A)** and assembly of ectosomes **(B)**. **(A)** shows the composition of the membrane and luminal cargos of an exosome. The components shown in the membrane are among the most abundant in these vesicles. The content shows numerous proteins, some of which bound to miRNAs. Other components are fluid factors such as cytokines and growth factors shown as arrows. ESCRT complexes participate of membrane growth. mRNAs and ns-RNAs are other nucleotides accumulated here from the cytosol. **(B)** illustrates an example of ectosome rapidly assembled from the initial microdomain of the plasma membrane. To the left, 1) shows an initial curvature, already associated to a first cargo, followed by its budding 2) and then by its fission dependent on ESCRT-III interaction with VPS 3). To the right 4) the ectosome is free, ready to navigate in the extracellular fluid. The ectosome yellow sequences shown in the **(B)** lumen correspond to those drown in the exosome lumen of **(A)**.

Membrane. The generation of ILV membrane processes are mostly governed by the Endosomal Sorting Complexes Required for Transport proteins, i.e. by ESCRT-0, -I and -II, working together with their associated protein factors to induce membrane curvature (Gruenberg, 2020; Pavlin and Hurley, 2020; Juan and Fürthauer, 2018a). ESCRT-III subunits operate assembled with Alix and other proteins. Their interaction with helical filaments of various forms of vacuolar protein sorting (VPS) including an ATPase, mediates membrane remodeling (Huber et al., 2020). Fission occurs when ESCRT-III is removed, leading to scission of the ILVs neck (Figure 2B) (Huber et al., 2020; Johnson et al., 2018; Tseng et al., 2021). The membrane of ILVs contain a peculiar lipid, LBPA, present together with many specific proteins in the unique MBV endocytic vacuoles (Gruenberg, 2020). Abundant are the small tetraspanins (predominant CD63), critically important during vesicle assembly with membrane and cargo protein trafficking. Additional proteins of lower concentration, including adhesion proteins, receptors, glycoproteins and metalloproteases, are present in ILV membranes (Figure 2A) (Meldolesi et al., 1978; Pizzirani et al., 2007). In a variety of cell types ILV loading within MVBs and the ensuing release of exosomes depend on sirtuin2, a deacetylase enzyme known to participate also in several other processes (Lee et al., 2019).

Cargos. Luminal cargos begin their accumulation in the initial ILV membrane protrusions. Among proteins, those concentrated in the lumen are typical of growing vesicles. Unconventional secretory proteins are also present, however at concentration lower than that of tetraspanins (Meldolesi, 2018). Small cytosolic proteins are also trapped within ILVs. In addition to proteins, cargos contain molecules of different nature: various types of RNA (mostly microRNAs, miRs, together with messenger RNAs, mRNAs, long non-coding RNAs, lncRNAs, and ribosome RNAs, rRNAs), small sequences of DNA, lipids and metabolic molecules (Figure 2A) (Yang and Gould, 2013; Ras-Carmona et al., 2021). Recent studies have shown ubiquitin and ubiquitin-like proteins to participate in protein post-translational modifications and in the control of protein complex composition (Chen et al., 2021; Padovani et al., 2022), thus contributing to their accumulation within ILVs (Yang and Gould, 2013; Chen et al., 2021; Ras-Carmona et al., 2021). Surface proteins of cargo are often anchored to the ILV membrane by myristoylation, palmitoylation or other sequences. The mechanisms of cargo accumulation remain unclear. Discoveries of a few years ago demonstrated the targeting and association of single proteins to the growing ILV lumen (Chen et al., 2021). Details have been clarified by the identification of proteins, such as endofin and arrestin-domain containing protein (ARRDC1), in the assembly of cargos (Ageta and Tsuchida, 2019; Kazan et al., 2021). Condensates of another protein of ample specificity, YBX1, have been shown to induce abundant liquid-liquid phase separations selectively recruiting a single miRNA, miR223 (Liu H. et al., 2021). The present hypothesis is that, in cargo development, other RNA-binding proteins undergo condensation. By such mechanism various proteins, together with important factors such as IL-1β and TNF-α, undergo a selective engulfment within ILVs before release by unconventional secretion. (Bello ‐ Gamboa et al., 2020).

Journey and exocytosis of MVBs. Once established, MVBs travel within the cell. In immune and other cells, actin reorganization induces convergence of the non-lysosome binding fraction to the microtubule-organizing center (Bello ‐ Gamboa et al., 2020). In response to various types of stimulation MVBs move, approaching the plasma membrane (Figure 1) (Calvo and Izquierdo, 2020). Understanding of these processes has been strengthened by the use of optical reporters associated with ILV markers (Verweij et al., 2018; Liu X.-M. et al., 2021). Upon tethering to specific sites (Davis et al., 2021), some heterogeneity in the molecules participating in MVB exocytosis has been reported among various cell types. The first Ras GTPase reported in the process has been Ras 11. Additional forms, such as Rab27a and Rab 27b, as well as the Rho, Rac, cdc42 family, have been reported to operate in many, but not in all types of cells (Hessvik and Llorente, 2018; Hyenne et al., 2018; Colombo et al., 2021). Concerning the fusion complex, the factor most frequently involved in various tissues and also in cancers (Peng et al., 2021) is the R-SNARE VAMP7 (vesicle-associated protein 7) together with the Q-SNARE SNAP23. Other R-SNAREs, such as VAMP3 and VAMP8, are also effective, however with lower frequency (Verweij et al., 2018; Zhao et al., 2022). Their ternary complex, established with SNAP23 associated to Syntaxin-4, induces the generation of enlarging pores, called invadopodia, key sites for MVB fusion with the plasma membrane and for the ensuing ILV release (Puri and Roche, 2006; Verweij et al., 2018; Colombo et al., 2021; Peng et al., 2021; Zhao et al., 2022). Q-SNAREs analogous to SNAP23, such as SNAP25, are ineffective in such fusion. Additional small GTPases involved include Ral, Rab (especially Rab35) and other Ras (Zhu et al., 2015; Yang et al., 2019). The integrated analysis of the various participants has revealed the role of non-coding RNAs (Yang et al., 2019) and G protein-coupled receptors. The latter, via their cAMP effect, promote the fusion via a SNAP23 phosphorylation occurring at the Ser 110 position (Verweij et al., 2018). SNARE dependence participates also in the unconventional secretion of important proteins such as *α*-synuclein (Zhao et al., 2022). The latter aspect will be presented in the following Section 5.

During the last few years, exocytosis and exosome secretion have been intensely investigated by a variety of techniques. The recent development of pH-dependent fluorescence microscopy has introduced the direct revelation of the processes. Fluorescent proteins of exosome membranes, such as CD63-pHfluorin, start emitting fluorescence upon exocytosis. The steps revealed by these approaches are numerous, from the efficacy and intracellular signaling of exogenous stimuli to the frequency, localization and machinery of exocytosis, up to the navigation of the released vesicles (Liu X.-M. et al., 2021; Gurung et al., 2021). The exosome localization studies have revealed various unexpected results. In lymphocytes the site of exocytosis is redistributed upon the establishment of immune synapses (Bello ‐ Gamboa et al., 2020). In epithelial cells, where the plasma membrane includes two distinct areas, MVB exocytosis addressed to the baso-lateral area are different from those addressed to the apical area. Differences have been demonstrated also between the two corresponding families of released exosomes (Colombo et al., 2021; Matsui et al., 2021). Heterogeneity is therefore a common property even of exosomes secreted by single cells.

### 3.2 Ectosomes

As already mentioned knowledge of ectosomes, more limited than that of ILV/exosomes, has been questioned for many years. This because the preparations employed were widely contaminated by membrane fragments of other origin; and because the studies of ectosomes were less numerous and less detailed compared to those of ILV/exosomes. Nevertheless, the information about ectosomes has grown, dependent in many cases on parallel studies about both exosomes and ectosomes (Van Niel et al., 2018; Meldolesi, 2018). Compared to exosomes, the intracellular life of ectosomes is much shorter. In cells stimulated by a variety of agents, such as ATP, ectosome generation by outward budding and pinching off of small plasma membrane microdomains starts within a few minutes (Van Niel et al., 2018) (Figure 2B). Regulation of ectosome generation depends on various factors. Cdc42, a small G protein of the Rho family, is a convergent node of multiple regulatory signals. The binding to its downstream effector, Ras GTPase-activating-like protein 1 (IQGAP1), is required for ectosome shedding (Wang et al., 2021; Dai et al., 2019). Additional stimulatory events are the up-regulation of RhoA, Rock and phosphorylated LINK1, a kinase that controls actin cytoskeleton dynamics. RhoA inhibitors suppress the production of ectosomes (Sun et al., 2021). Subsequent developments are supported by involvement of ESCRTs and their associated proteins analogous, but not identical, to those of ILVs (Gruenberg, 2020; Pavlin and Hurley, 2020; Juan and Fürthauer, 2018a; Huber et al., 2020). At least two complexes activate typical processes, i.e. their membrane dynamics involves outward budding and fission of corresponding plasma membrane microdomains. (Askenase, 2021; Pavlin and Hurley, 2020). ESCRT-III, followed by appropriate ATPase, governs the increased curvature, with ensuing narrowing of the neck followed by final scission (Figure 2B) (Johnson et al., 2018; Huber et al., 2020; Tseng et al., 2021; Wang et al., 2021). Regulation of further processes depends also on protein phosphorylation and calmodulin activation (Ni et al., 2020). Based on their properties, ectosomes and their molecules play a critical role in the regulation of cellular biology (Lv et al., 2019).

Membranes, Cargo, Release. The microdomains involved in the generations of ectosomes exhibit differences with respect to the rest of the plasma membrane. Their asymmetric phospholipid layers are rapidly rearranged. Several membrane proteins are analogous, but not identical to those of ILVs. For example, the most abundant tetraspanin in ectosomes is not the CD63 of ILVs but CD9 (Mathieu et al., 2021). Proteins of plasma membrane are present in ectosome membranes during and after their generation, however at low concentration.

Knowledge about ectosome cargo is limited. Accumulation of proteins by high affinity binding to miRNAs are processes known within exosomes (Yang and Gould, 2013; Chen et al., 2021). They might occur also within the ectosome lumen. Among such proteins is ARRDC1, an adaptor of ubiquitin ligases, involved in the regulation of ectosome generation and release (Anand et al., 2018). Loading of ectosome cargos with both RNA-binding proteins and miRNAs are supported by the LC3-conjugated machinery, an example of vesicle/autophagy interaction (Leidal et al., 2020). The ectosome cargo formation is regulated by caveolin-1, a structural protein as abundant as in plasma membrane caveolae. In contrast in exosome cargos caveolin-1 is not abundant (Ni et al., 2020) In addition, the ectosome cargos contain IL-1β, various cytokines and factors of the TNF family, together with other proteins of unconventional secretion (Cohen et al., 2020). Upon release from the plasma membrane, ectosomes coincide to EVs characterized by their large size and specific extracellular properties. In epithelial cells, plasma membrane areas induce two distinct types of such EVs, characterized by different functional roles (Colombo et al., 2021).

## 4 EVs: Origin, Navigation and Fusion With Target Cells

Contrary to some conventional beliefs, EVs are not membrane fragments released as a result of cell leakage. They are two types of extracellular vesicles, secreted by all cells and providing routes of intercellular communication. In fact, they transmit *in vitro* and *in vivo* biological messages between cells, with activation of specific signal transductions in their target cells. As a consequence, EVs are important tools active for unconventional secretion. Upon reaching their extracellular space, EVs start their navigation. An unexpected property of such activity is the vastness of its traffic, which is not restricted to the space adjacent to their cells of origin but is almost unlimited in the whole body. EVs are in fact the only membrane-bound structures to which the blood-brain barrier and various types of intercellular junctions are largely permeable. This property explains the EV circulation among organs and in the fluid spaces of the body, most important being the central spinal fluid and the blood plasma. Summing up, the wide navigation is essential for EV interaction and fusion with target cells. Their signal transduction cascades are activated at all distances of intercellular communications.

The specific properties of the various EVs are mostly due to the elaborate assembly of their precursors. During their biogenesis, the bending away and the ensuing budding and fission of membranes are established with the contribution of ESCRT complexes (Juan and Fürthauer, 2018; Cocozza et al., 2020; Pavlin and Hurley, 2020; Askenase 2021; Lim et al., 2021; Sun et al., 2021). Recent evidence has demonstrated that ESCRT complexes contribute also to a variety of EV processes, including their membrane specificity and the integrated structure of their cargos (Juan and Fürthauer, 2018). The membrane composition is complex. In addition to members of the teraspanin family, tthey include glycoproteins, adhesion molecules, various lipids and also receptors (Figure 2A), important for binding to other vesicles and target cells. Their cargos include long-term proteins often bound to miRNAs, which are known to possess sorting sequences involved in the regulation of their secretion (Garcia-Martin et al., 2021). Additional components include other types of non-coding RNAs and coding mRNAs together with lipids, enzymes, cytokines, growth factors and various proteins of unconventional secretion, most often originated from the cytoplasm (Boilard, 2018; Greening and Simpson, 2018; Garcia-Martin et al., 2021). Among phospholipid components, abundant are the arachidonic acids and other polyunsaturated eicosanoids (Juan and Fürthauer, 2018). Release of EVs to the extracellular space depends on the functional state of their cells of origin (Hessvik and Llorente, 2018; Cohen et al., 2020; Sun et al., 2021). Additional mechanisms known to regulate the EV generation and function depend on the media of their culture. For example, addition of fetal bovine serum, widely used commercially, induces negative effects, whereas with various growth factors and also with glucose the induced effects are positive (Bost et al., 2021; Guan et al., 2021).

Comparative analysis of the EVs derived from exosomes and ectosomes have revealed interesting aspects of heterogeneity. Proteins of important families, from their membranes (tetraspanins) and lumena (proteins associated to transport and fusion) are present in both types of EVs, however with differences in their levels and components (Pizzirani et al., 2007; Van Niel et al., 2018; Bost et al., 2021; Guan et al., 2021; Mathieu et al., 2021). Differences have been reported also for weakly expressed proteins, some of which heterogeneous also among EVs from distinct subtypes of cellular vesicles (Greening and Simpson, 2018; Gurunathan et al., 2021). Exosomes and ectosomes differ also in metabolites, a property relevant for navigation fluids, including blood plasma (Bost et al., 2021; Gurunathan et al., 2021; Lim et al., 2021). In addition, recent evidence has demonstrated the ability of EVs to establish surface protein-protein interactions. It appears, therefore, that at least a fraction of EVs tends to show functionally integrated complexes (Leidal et al., 2020; Levy et al., 2021; Nikoloff et al., 2021; Razzauti and Laurent, 2021). At present, populations of single EV type can be isolated by various techniques based on distinct approaches, including monoclonal antibodies (Levy et al., 2021; Lim et al., 2021). The state of knowledge and techniques are already advanced. Additional developments are expected for the future including their markers and signatures, useful for the identification of subtype-specific EVs and the unconventional secretion of their proteins (Garcia-Martin et al., 2021; Levy et al., 2021; Nikoloff et al., 2021; Razzauti and Laurent, 2021).

**FIGURE 3:**
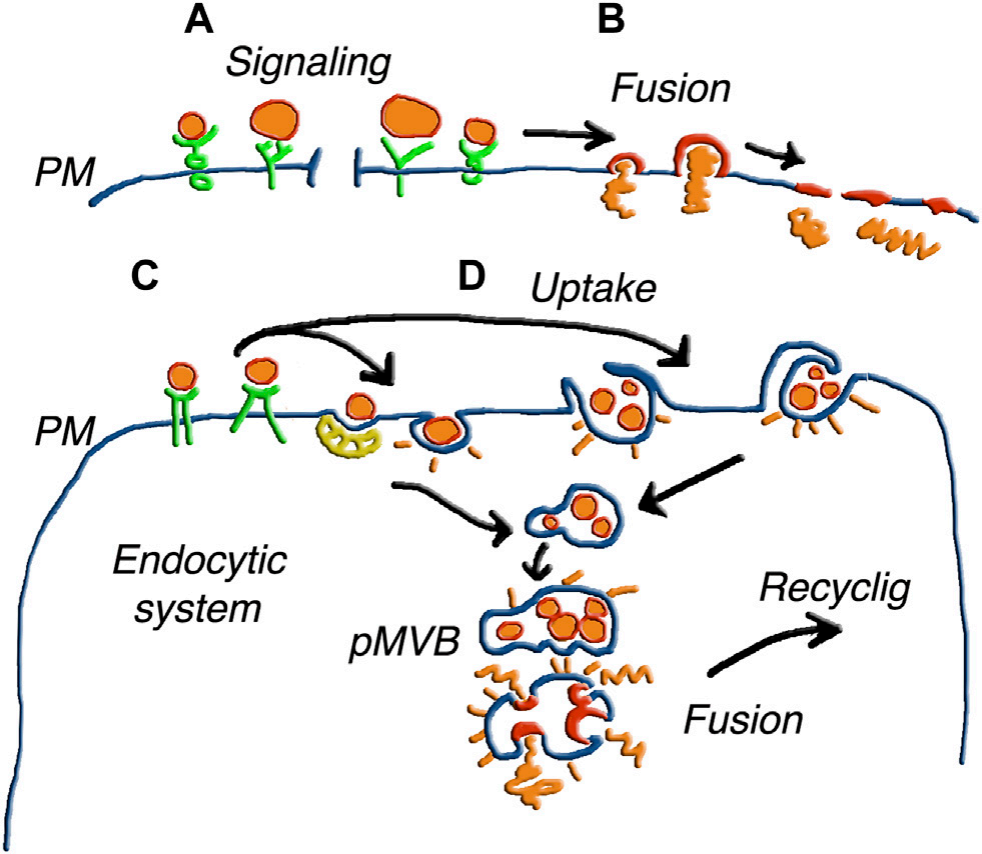
Various forms of EV fusion with target cells. The different size of the EVs illustrates the parallel processes of ectosomes and exosomes. **(A)** shows vesicles inducing only signaling upon binding to receptors. In **(B)** the fusion of single vesicles followed by the integration of the membrane the plasma membrane (PM), induces release of cargo to the cytoplasm. In **(C)** the EVs, bound to receptors such as those in **(A)**, are internalized in an endosomal cisterna, and the same occurs in **(D)** for EV groups internalized by phagocytosis or macro-pinocytosis. Upon internalization, the EVs of **(C,D)** are accumulated within pseudo multi-vesicular bodies (pMVB). Elimination of these structures by ectosomal block and possible fusion with lysosomes do not appear in this Figure. Alternatively, the EV membranes fuse with endosome membrane and the cargos are released in the depth of the cytoplasm. The possible integration of molecules from fused EVs with molecules generated locally to participate in a new generation of EVs is suggested by the word Recycling. Figure reproduced with permission from Meldolesi (2018).

After navigation in their extracellular fluid, EVs can undergo fusion with target cells, not only in the proximity but also at large distances from their cells of origin. Such fusions need to be efficient and specific, inducing transfer of their cargos to target cells. The first step is a tethering, which is essential, established between vesicles and the surface of target cells. In fact, block of tethering results in the prevention of all fusions (Guan et al., 2021; Gurunathan et al., 2021). On the other hand, tethering is not followed by fusion in all cases. The ensuing interactions are not always of the same type. The specific binding of a vesicle agonist to its receptor at the cell surface can be transient followed by generation of intracellular signals. In many cases, however, binding is followed by insertion of the vesicle membrane in the plasma membrane, with enlargement of the cell surface area and cargo discharged into the cell cytoplasm (Levy et al., 2021; An et al., 2021). Upon the introduction of new techniques to reveal the progressive changes of the EV protein distribution, the membrane and cargo events have been intensively investigated (Lim et al., 2021; Bost et al., 2021). Where does the EV fusion occur in target cells? In only a fraction of cases the process occurs at the surface, and cargos are discharged through the plasma membrane (Figure 3) (Somiya and Kuroda, 2021; Perissinotto et al., 2021; Song et al., 2021; Hung and Leonard, 2016). In many other cases fusion, preceded by the internalization of EV in the endocytic system, occurs in a moderately acidic environment (Figure 3) (Joshi et al., 2020; Somiya, 2020). A possible consequence of this pathway is the capture of EVs by the endo/lyso system, with limited escape of their cargos to the cytoplasm. However, the disruption of discharged cargo molecules is not always the case. Rather, many proteins remain intact and functional, as it happens with receptors still active upon their discharge (Hung and Leonard, 2016; Somiya, 2020; Levy et al., 2021). During and after such fusions also target cells release vesicles of their origin, containing at least part of the components received by their EV fusion. A process of this type is usually called recycling (Somiya, 2020). In conclusion, secretion pathways of EVs and their components appear more complex than previously expected. The problem is further discussed in the following Section focused exclusively on unconventional secretion.

## 5 Relevant Examples of Unconventional Protein Secretion

Interest about EVs is growing depending on their properties such heterogeneity, navigation, fusion and recycling. In previous Sections unconventional secretion of proteins has been described, however only marginally. Here their presentation is expanded, focusing on the physiology of well-know examples. At present, in fact, unconventional protein secretion is not interesting only for basic physiology. It is relevant in diagnoses, therapies and clinical applications of diseases in the brain, heart and vessels, bones, and other tissues (Ng and Tang, 2016; Joshi et al., 2020; Gurunathan et al., 2021; He et al., 2021). In the present review, however, no space is available to deal with the EV role in these diseases. News about them will be reported in a future review to be published elsewhere.

Mechanisms underlying unconventional secretion have been deciphered during the last several years (Ng and Tang, 2016; Cohen et al., 2020). Recent developments about immune modulations, cell signaling, growth, redox control, as well as moonlighting activities, have started to be identified and characterized (Cohen et al., 2020; Sitia and Rubartelli, 2020). High interest for EV crosstalk has been demonstrated with other structures and functions, such as autophagy and inflammasomes. Autophagy is best known for its role in organelle and protein turnover. In addition their machinery, together with MVBs, participates in ILV assembly (Bebelman et al., 2020; Raudenska et al., 2021). Machinery and MVBs often fuse with lysosomes, and in these cases they are often digested. In other cases, however, changes occur during MVB traffic towards the plasma membrane. Upon their integration in EV structure, autophagy components follow the EV pathway, from their exocytosis to navigation and cell fusion, activating the unconventional accumulation of important proteins in target cells (Hassanpour et al., 2020; Raudenska et al., 2021). Among such proteins is tau, known to play a critical role in brain diseases such as Alzheimer’s and tau diseases. Its autophagy crosstalk with the two types of EV appears relevant for unconventional secretion involving neurons, astrocytes and microglia (Brunello et al., 2020; Jiang and Bhaskar, 2020). Another example of crosstalk concerns SCAMP5 (Secretory Carrier Membrane Protein 5), an inhibitor of autophagosome fusion with lysosomes. Its increased activity promotes a fragmentation of GC with block of its conventional and increased unconventional secretion (Yang et al., 2017). This unexpected switch of the secretory processes is relevant because it results in physiological changes including the metabolism of important proteins such as *α*-synuclein (Yang et al., 2017; Zhao et al., 2022). Interestingly, once *α*-synuclein metabolism is switched away from conventional secretion, it can be addressed not only to EVs but also to other unconventional pathways including autophagosomes and lysosomes (Zhao et al., 2022). In addition to brain physiology, unconventional secretion is important for inter-neuronal transmission (Yang et al., 2017; Bieri et al., 2018). Moreover, autophagic crosstalk concerns two proteins unconventionally secreted by teratocyte cells of insects upon their accumulation within exosomes (Salvia et al., 2019).

The most important form of vesicle crosstalk deals however with inflammasomes. The latter are large protein complexes assembled by variously recognized receptors together with pathogen-associated or damage-associated protein patterns, both activated by caspase-1 (Dai et al., 2019). Generation of various exosomes by inflammations results in the activation of several tasks including unconventional secretion of IL-1β, IL-18 and many other proteins. Moreover, exosomes can induce promotion or inhibition of various types of inflammasome (Bieri et al., 2018; Salvia et al., 2019). Finally, crosstalk of a peculiar inflammation with ectosomes, rather than exosomes, regulates the expression and unconventional secretion of an important protein, FADD, involved in a number of important processes: cell death, proliferation, immunity and inflammation (Cypryk et al., 2018; Noonin and Thongboonkerd, 2021). Thus, EVs of the two families, combined to various inflammasomes, induce distinct effects of critical importance for unconventional secretion (Bieri et al., 2018; Cypryk et al., 2018; Salvia et al., 2019; Noonin and Thongboonkerd, 2021).

Secretion of other proteins can occur by either conventional or unconventional pathways depending on their structure or binding to other component. This is the case of the cytokine TNF, which is switched off by the conventional secretion of ATP. In contrast, ATP induces TNF accumulation within ectosomes followed by exocytosis, navigation and fusion, thus establishing cell-to-cell connections (Noonin and Thongboonkerd, 2021). Another protein, the proinflammatory transmembrane protease TACE, when expressed upon its tyrosine phosphorylation, induces loss of a trafficking factor followed by its translocation into EVs and secretion via their unconventional pathway (Zhao et al., 2019). On the other hand, galectin-3 was found to include in its amino terminus a highly conserved tetrapeptide motif necessary for its direct binding to Tsg101, an associated protein of the ESCRT complex. Such motif is necessary for galectin to undergo unconventional secretion with exosomes. Mutations of its tetrapeptide prevents secretion of galectin-3 (Bänfer et al., 2018).

## 6 Conclusion

The unconventional secretion emerging from the present review is unique. As emphasized in Section 1, its discovery was based on the recognition of properties distinct from those of conventional secretion. Here attention has been focused on the two unconventional secretory EVs, the small exosomes and the larger ectosomes. In the last Section the properties of the two EVs are summarized together, including processes that in previous Sections are presented separately.

The membranes and cargos of the two types of EV are largely, but not completely, different from the membranes and cytoplasm of their original cells. Various distinctions exist between these two types of vesicles, concerning not only their size but also their molecular composition (Meldolesi, 2018; Van Niel et al., 2018; Royo et al., 2020; Raudenska et al., 2021), intracellular life (Rabouille, 2017; Van Niel et al., 2018; Gurung et al., 2021; Raudenska et al., 2021), and release processes (Van Niel et al., 2018; Shtam et al., 2020; Gurung et al., 2021; Ras-Carmona et al., 2021; Raudenska et al., 2021). A major distinction of EVs, however, exists with respect to the vesicles and granules of conventional secretion. From such organelles only cargos are secreted, most often into the extracellular space. During their release the membranes are first integrated in the plasma membrane and then recycled by endocytosis to the cytoplasm of secretory cells. In contrast the EVs, upon their release and during navigation, maintain their whole structure, composed by both membranes and cargos. Upon their fusion to specific target cells, their membranes are integrated into the plasma or endocytic membranes, while the cargos are discharged and diffuse in the cytoplasm (Bost et al., 2021; Guan et al., 2021). Depending on their properties, the intracellular distribution of the received cargo proteins varies: a l fraction goes to the nucleus, others to the plasma membrane/intracellular membranes, or remain in the cytosol (Somiya and Kuroda, 2021). In each animal, received cargo proteins recycle within re-assembled EVs, circulating along their pathways distributed following dynamic, possibly interconnected networks. Based on the present knowledge, the distinction of the two EVs with respect to conventional secretory organelles has been hypothesized to depend on their ancient evolutionary origin (Askenase, 2021).

The results of EV fusions induce many operational effects, distinct from those of conventional secretion, including epigenetic mechanisms, widely spread among cells. Unconventionally secreted proteins presented in the review include tau, *α*-synuclein, SCAMP, FADD, interleukins (Yang et al., 2017; Bieri et al., 2018; Cypryk et al., 2018; Salvia et al., 2019; Brunello et al., 2020; Hassanpour et al., 2020; Jiang and Bhaskar, 2020; Sitia and Rubartelli, 2020). A list of highly relevant protein secretion can switch from conventional to unconventional and vice-versa, depending on the cells involved and their specific environmental conditions (Yang et al., 2017; Bieri et al., 2018). Moreover, interaction with autophagy can lead to fusion including machinery molecules (Hassanpour et al., 2020; Raudenska et al., 2021); and crosstalk of EVs with inflammasomes induces unconventional secretory proteins dependent on immune responses (Cypryk et al., 2018; Mouasni et al., 2019; Noonin and Thongboonkerd, 2021). In conclusion, the wide circulation of EVs can operate by biological and also medical processes dependent on critical molecules that do not diffuse independently through intercellular fluids but exchanges by reciprocal fusion of EVs between cells.

Compared to conventional secretion, knowledge of unconventional secretion is still limited, however it is growing, especially in the disease areas already intensely investigated (see for example the reviews Gonzalez et al., 2020; Raffaele et al., 2020; Ganesan and Cai, 2021). In the near future, therefore, the role of unconventional protein secretion will become more and more relevant in key areas, from physiology to medicine and especially to clinical practice.

## References

[B1] Ageta H. Tsuchida K. (2019). Post-translational Modification and Protein Sorting to Small Extracellular Vesicles Including Exosomes by Ubiquitin and UBLs. Cell Mol. Life Sci. 76, 4829–4848. 10.1007/s00018-019-03246-7 31363817 PMC11105257

[B2] Akatsuka H. Kawai E. Omori K. Shibatani T. (1995). The Three Genes lipB, lipC, and lipD Involved in the Extracellular Secretion of the Serratia marcescens Lipase Which Lacks an N-Terminal Signal Peptide. J. Bacteriol. 177, 6381–6389. 10.1128/jb.177.22.6381-6389.1995 7592412 PMC177487

[B3] An S. J. Rivera-Molina F. Anneken A. Xi Z. McNellis B. Polejaev V. I. Toomre D. (2021). An Active Tethering Mechanism Controls the Fate of Vesicles. Nat. Commun. 12, 5434. 10.1038/s41467-021-25465-y 34521845 PMC8440521

[B4] Anand S. Foot N. Ang C. S. Gembus K. M. Keerthikumar S. Adda C. G. Mathivanan S. Kumar S. (2018). Arrestin‐Domain Containing Protein 1 (Arrdc1) Regulates the Protein Cargo and Release of Extracellular Vesicles. Proteomics 18, 1800266. 10.1002/pmic.201800266 30035390

[B5] Askenase P. W. (2021). Ancient Evolutionary Origin and Properties of Universally Produced Natural Exosomes Contribute to Their Therapeutic Superiority Compared to Artificial Nanoparticles. Ijms 22, 1429. 10.3390/ijms22031429 33572657 PMC7866973

[B6] Bänfer S. Schneider D. Dewes J. Strauss M. T. Freibert S.-A. Heimerl T. Maier U. G. Elsässer H.-P. Jungmann R. Jacob R. (2018). Molecular Mechanism to Recruit Galectin-3 into Multivesicular Bodies for Polarized Exosomal Secretion. Proc. Natl. Acad. Sci. U.S.A. 115, E4396–E4405. 10.1073/pnas.1718921115 29686075 PMC5948969

[B7] Bebelman M. P. Bun P. Huveneers S. van Niel G. Pegtel D. M. Verweij F. J. (2020). Real-time Imaging of Multivesicular Body-Plasma Membrane Fusion to Quantify Exosome Release from Single Cells. Nat. Protoc. 15, 102–121. 10.1038/s41596-019-0245-4 31836866

[B8] Bello‐Gamboa A. Velasco M. Moreno S. Herranz G. Ilie R. Huetos S. Dávila S. Sánchez A. Bernardino De La Serna J. Calvo V. Izquierdo M. (2020). Actin Reorganization at the Centrosomal Area and the Immune Synapse Regulates Polarized Secretory Traffic of Multivesicular Bodies in T Lymphocytes. J. Extracell. Vesicles 9, 1759926. 10.1080/20013078.2020.1759926 32939232 PMC7480611

[B9] Bieri G. Gitler A. D. Brahic M. (2018). Internalization, Axonal Transport and Release of Fibrillar Forms of Alpha-Synuclein. Neurobiol. Dis. 109, 219–225. 10.1016/j.nbd.2017.03.007 28323023 PMC5600643

[B10] Boilard E. (2018). Thematic Review Series: Exosomes and Microvesicles: Lipids as Key Components of Their Biogenesis and Functions Extracellular Vesicles and Their Content in Bioactive Lipid Mediators: More Than a Sack of microRNA. J. Lipid Res. 59, 2037–2046. 10.1194/jlr.R084640 29678959 PMC6210911

[B11] Bost J. P. Saher O. Hagey D. Mamand D. R. Liang X. Zheng W. Corso G. Gustafsson O. Görgens A. Smith C. E. Zain R. El Andaloussi S. Gupta D. (2021). Growth Media Conditions Influence the Secretion Route and Release Levels of Engineered Extracellular Vesicles. Adv. Healthc. Mater. 11, 2101658. 10.1002/adhm.202101658 PMC11469210 34773385

[B12] Brunello C. A. Merezhko M. Uronen R.-L. Huttunen H. J. (2020). Mechanisms of Secretion and Spreading of Pathological Tau Protein. Cell. Mol. Life Sci. 77, 1721–1744. 10.1007/s00018-019-03349-1 31667556 PMC7190606

[B13] Calvo V. Izquierdo M. (2020). Inducible Polarized Secretion of Exosomes in T and B Lymphocytes. Ijms 21, 2631. 10.3390/ijms21072631 PMC7177964 32290050

[B14] Caro L. G. Palade G. E. (1964). Protein Synthesis, Storage, and Discharge in the Pancreatic Exocrine Cell. J. Cell Biol. 20, 473–495. 10.1083/jcb.20.3.473 14128049 PMC2106415

[B15] Chen Y. Zhao Y. Yin Y. Jia X. Mao L. (2021). Mechanism of Cargo Sorting into Small Extracellular Vesicles. Bioengineered 12, 8186–8201. 10.1080/21655979.2021.1977767 34661500 PMC8806638

[B16] Cocozza F. Grisard E. Martin-Jaular L. Mathieu M. Théry C. (2020). SnapShot: Extracellular Vesicles. Cell 182, 262–262e1. 10.1016/j.cell.2020.04.054 32649878

[B17] Cohen M. J. Chirico W. J. Lipke P. N. (2020). Through the Back Door: Unconventional Protein Secretion. Cell Surf. 6, 100045. 10.1016/j.tcsw.2020.100045 33225116 PMC7666356

[B18] Colombo F. Casella G. Podini P. Finardi A. Racchetti G. Norton E. G. Cocucci E. Furlan R. (2021). Polarized Cells Display Asymmetric Release of Extracellular Vesicles. Traffic 22, 98–110. 10.1111/tra.12775 33314523

[B19] Corbeil D. Santos M. F. Karbanová J. Kurth T. Rappa G. Lorico A. (2020). Uptake and Fate of Extracellular Membrane Vesicles: Nucleoplasmic Reticulum-Associated Late Endosomes as a New Gate to Intercellular Communication. Cells 9, 1931. 10.3390/cells9091931 PMC7563309 32825578

[B20] Cypryk W. Nyman T. A. Matikainen S. (2018). From Inflammasome to Exosome-Does Extracellular Vesicle Secretion Constitute an Inflammasome-dependent Immune Response? Front. Immunol. 9, 2188. 10.3389/fimmu.2018.02188 30319640 PMC6167409

[B21] Dai H. Zhang S. Du X. Zhang W. Jing R. Wang X. Pan L. (2019). RhoA Inhibitor Suppresses the Production of Microvesicles and Rescues High Ventilation Induced Lung Injury. Int. Immunopharmacol. 72, 74–81. 10.1016/j.intimp.2019.03.059 30959374

[B22] Davis L. J. Bright N. A. Edgar J. R. Parkinson M. D. J. Wartosch L. Mantell J. Peden A. A. Luzio J. P. (2021). Organelle Tethering, Pore Formation and SNARE Compensation in the Late Endocytic Pathway. J. Cell Sci. 134, jcs255463. 10.1242/jcs.255463 34042162 PMC8186482

[B23] Ganesan D. Cai Q. (2021). Understanding Amphisomes. Biochem. J. 478, 1959–1976. 10.1042/BCJ20200917 34047789 PMC8935502

[B24] Garcia-Martin R. Wang G. Brandão B. B. Zanotto T. M. Shah S. Kumar Patel S. Schilling B. Kahn C. R. (2021). MicroRNA Sequence Codes for Small Extracellular Vesicle Release and Cellular Retention. Nature 601, 446–451. 10.1038/s41586-021-04234-3 34937935 PMC9035265

[B25] Gonzalez C. D. Resnik R. Vaccaro M. I. (2020). Secretory Autophagy and its Relevance in Metabolic and Degenerative Disease. Front. Endocrinol. 11, 266. 10.3389/fendo.2020.00266 PMC7232537 32477265

[B26] Greening D. W. Simpson R. J. (2018). Understanding Extracellular Vesicle Diversity - Current Status. Expert Rev. Proteomics 15, 887–910. 10.1080/14789450.2018.1537788 30326765

[B27] Gruenberg J. (2020). Life in the Lumen: The Multivesicular Endosome. Traffic 21, 76–93. 10.1111/tra.12715 31854087 PMC7004041

[B28] Guan F. Xiang X. Xie Y. Li H. Zhang W. Shu Y. Wang J. Qin W. (2021). Simultaneous Metabolomics and Proteomics Analysis of Plasma-Derived Extracellular Vesicles. Anal. Methods 13, 1930–1938. 10.1039/d1ay00060h 33913941

[B29] Gurunathan S. Kang M.-H. Qasim M. Khan K. Kim J.-H. (2021). Biogenesis, Membrane Trafficking, Functions, and Next Generation Nanotherapeutics Medicine of Extracellular Vesicles. Ijn 16, 3357–3383. 10.2147/IJN.S310357 34040369 PMC8140893

[B30] Gurung S. Perocheau D. Touramanidou L. Baruteau J. (2021). The Exosome Journey: from Biogenesis to Uptake and Intracellular Signalling. Cell Commun. Signal 19, 47. 10.1186/s12964-021-00730-1 33892745 PMC8063428

[B31] Hassanpour M. Rezabakhsh A. Rezaie J. Nouri M. Rahbarghazi R. (2020). Exosomal Cargos Modulate Autophagy in Recipient Cells via Different Signaling Pathways. Cell Biosci. 10, 92. 10.1186/s13578-020-00455-7 32765827 PMC7395405

[B32] He J. Ren W. Wang W. Han W. Jiang L. Zhang D. Guo M. (2021). Exosomal Targeting and its Potential Clinical Application. Drug Deliv. Transl. Res. 10.1007/s13346-021-01087-1 PMC9458566 34973131

[B33] Hessvik N. P. Llorente A. (2018). Current Knowledge on Exosome Biogenesis and Release. Cell. Mol. Life Sci. 75, 193–208. 10.1007/s00018-017-2595-9 28733901 PMC5756260

[B34] Huber S. T. Mostafavi S. Mortensen S. A. Sachse C. (2020). Structure and Assembly of ESCRT-III Helical Vps24 Filaments. Sci. Adv. 6, eaba4897. 10.1126/sciadv.aba4897 32875105 PMC7438092

[B35] Hung M. E. Leonard J. N. (2016). A Platform for Actively Loading Cargo RNA to Elucidate Limiting Steps in EV-Mediated Delivery. J. Extracell. Vesicles 5, 31027. 10.3402/jev.v5.31027 27189348 PMC4870355

[B36] Hyenne V. Labouesse M. Goetz J. G. (2018). The Small GTPase Ral Orchestrates MVB Biogenesis and Exosome Secretion. Small GTPases 9, 445–451. 10.1080/21541248.2016.1251378 27875100 PMC6204988

[B37] Jiang S. Bhaskar K. (2020). Degradation and Transmission of Tau by Autophagic-Endolysosomal Networks and Potential Therapeutic Targets for Tauopathy. Front. Mol. Neurosci. 13, 586731. 10.3389/fnmol.2020.586731 33177989 PMC7596180

[B38] Johnson D. S. Bleck M. Simon S. M. (2018). Timing of ESCRT-III Protein Recruitment and Membrane Scission during HIV-1 Assembly. Elife 7, e36221. 10.7554/eLife.36221 29972351 PMC6080951

[B39] Joshi B. S. de Beer M. A. Giepmans B. N. G. Zuhorn I. S. (2020). Endocytosis of Extracellular Vesicles and Release of Their Cargo from Endosomes. ACS Nano 14, 4444–4455. 10.1021/acsnano.9b10033 32282185 PMC7199215

[B40] Juan T. Fürthauer M. (2018). Biogenesis and Function of ESCRT-dependent Extracellular Vesicles. Seminars Cell & Dev. Biol. 74, 66–77. 10.1016/j.semcdb.2017.08.022 28807885

[B42] Karim M. A. Samyn D. R. Mattie S. Brett C. L. (2018). Distinct Features of Multivesicular Body-Lysosome Fusion Revealed by a New Cell-free Content-Mixing Assay. Traffic 19, 138–149. 10.1111/tra.12543 29135058

[B43] Kazan J. M. Desrochers G. Martin C. E. Jeong H. Kharitidi D. Apaja P. M. Roldan A. St. Denis N. Gingras A.-C. Lukacs G. L. Pause A. (2021). Endofin Is Required for HD-PTP and ESCRT-0 Interdependent Endosomal Sorting of Ubiquitinated Transmembrane Cargoes. iScience 24, 103274. 10.1016/j.isci.2021.103274 34761192 PMC8567383

[B44] Kowal J. Arras G. Colombo M. Jouve M. Morath J. P. Primdal-Bengtson B. Dingli F. Loew D. Tkach M. Théry C. (2016). Proteomic Comparison Defines Novel Markers to Characterize Heterogeneous Populations of Extracellular Vesicle Subtypes. Proc. Natl. Acad. Sci. U.S.A. 113, E968–E977. 10.1073/pnas.1521230113 26858453 PMC4776515

[B45] Lee B. R. Sanstrum B. J. Liu Y. Kwon S.-H. (2019). Distinct Role of Sirtuin 1 (SIRT1) and Sirtuin 2 (SIRT2) in Inhibiting Cargo-Loading and Release of Extracellular Vesicles. Sci. Rep. 9, 20049. 10.1038/s41598-019-56635-0 31882861 PMC6934595

[B46] Leidal A. M. Huang H. H. Marsh T. Solvik T. Zhang D. Ye J. Kai F. Goldsmith J. Liu J. Y. Huang Y.-H. Monkkonen T. Vlahakis A. Huang E. J. Goodarzi H. Yu L. Wiita A. P. Debnath J. (2020). The LC3-Conjugation Machinery Specifies the Loading of RNA-Binding Proteins into Extracellular Vesicles. Nat. Cell Biol. 22, 187–199. 10.1038/s41556-019-0450-y 31932738 PMC7007875

[B47] Levy D. Do M. A. Zhang J. Brown A. Lu B. (2021). Orchestrating Extracellular Vesicle with Dual Reporters for Imaging and Capturing in Mammalian Cell Culture. Front. Mol. Biosci. 8, 680580. 10.3389/fmolb.2021.680580 34222335 PMC8249585

[B48] Lim H. J. Yoon H. Kim H. Kang Y.-W. Kim J.-E. Kim O. Y. Lee E.-Y. Twizere J.-C. Rak J. Kim D.-K. (2021). Extracellular Vesicle Proteomes Shed Light on the Evolutionary, Interactive, and Functional Divergence of Their Biogenesis Mechanisms. Front. Cell Dev. Biol. 9, 734950. 10.3389/fcell.2021.734950 34660591 PMC8517337

[B49] Liu H. Liu S. Xiao Y. Song W. Li H. Ho L. W. C. Shen Z. Choi C. H. J. (2021). A pH-Reversible Fluorescent Probe for *In Situ* Imaging of Extracellular Vesicles and Their Secretion from Living Cells. Nano Lett. 21, 9224–9232. 10.1021/acs.nanolett.1c03110 34724785

[B50] Liu X.-M. Ma L. Schekman R. (2021). Selective Sorting of microRNAs into Exosomes by Phase-Separated YBX1 Condensates. Elife 10, e71982. 10.7554/eLife.71982 34766549 PMC8612733

[B51] Lv Y. Tan J. Miao Y. Zhang Q. (2019). The Role of Microvesicles and its Active Molecules in Regulating Cellular Biology. J. Cell Mol. Med. 23, 7894–7904. 10.1111/jcmm.14667 31559684 PMC6850934

[B52] Mathieu M. Névo N. Jouve M. Valenzuela J. I. Maurin M. Verweij F. J. Palmulli R. Lankar D. Dingli F. Loew D. Rubinstein E. Boncompain G. Perez F. Théry C. (2021). Specificities of Exosome versus Small Ectosome Secretion Revealed by Live Intracellular Tracking of CD63 and CD9. Nat. Commun. 12, 4389. 10.1038/s41467-021-24384-2 34282141 PMC8289845

[B53] Matsui T. Osaki F. Hiragi S. Sakamaki Y. Fukuda M. (2021). ALIX and Ceramide Differentially Control Polarized Small Extracellular Vesicle Release from Epithelial Cells. EMBO Rep. 2222, e51475. 10.15252/embr.202051475 PMC8097368 33724661

[B54] Meldolesi J. Borgese N. De Camilli P. Ceccarelli B. (1978). “Cytoplasmic Membranes and the Secretory Process,” in Membrane Fusion. Editors Poste G. Nicolson G.L. (Amsterdam: Elsevier/North-Holland Biomed Press), 509–627.

[B55] Meldolesi J. (2018). Exosomes and Ectosomes in Intercellular Communication. Curr. Biol. 28, R435–R444. 10.1016/j.cub.2018.01.059 29689228

[B56] Mouasni S. Gonzalez V. Schmitt A. Bennana E. Guillonneau F. Mistou S. Avouac J. Ea H. K. Devauchelle V. Gottenberg J.-E. Chiocchia G. Tourneur L. (2019). The Classical NLRP3 Inflammasome Controls FADD Unconventional Secretion through Microvesicle Shedding. Cell Death Dis. 10, 190. 10.1038/s41419-019-1412-9 30804327 PMC6389912

[B57] Müller M. Ibrahimi I. Chang C. N. Walter P. Blobel G. (1982). A Bacterial Secretory Protein Requires Signal Recognition Particle for Translocation across Mammalian Endoplasmic Reticulum. J. Biol. Chem. 257, 11860–11863. 10.1016/s0021-9258(18)33642-1 6749848

[B58] Ng F. Tang B. L. (2016). Unconventional Protein Secretion in Animal Cells. Methods Mol. Biol. 1459, 31–46. 10.1007/978-1-4939-3804-9_2 27665549

[B59] Ni K. Wang C. Carnino J. M. Jin Y. (2020). The Evolving Role of Caveolin-1: a Critical Regulator of Extracellular Vesicles. Med. Sci. 8, 46. 10.3390/medsci8040046 PMC7712126 33158117

[B60] Nickel W. Rabouille C. (2009). Mechanisms of Regulated Unconventional Protein Secretion. Nat. Rev. Mol. Cell Biol. 10, 148–155. 10.1038/nrm2617 19122676

[B61] Nikoloff J. M. Saucedo-Espinosa M. A. Kling A. Dittrich P. S. (2021). Identifying Extracellular Vesicle Populations from Single Cells. Proc. Natl. Acad. Sci. U.S.A. 118, e2106630118. 10.1073/pnas.2106630118 34518226 PMC8463870

[B62] Noonin C. Thongboonkerd V. (2021). Exosome-inflammasome Crosstalk and Their Roles in Inflammatory Responses. Theranostics 11, 4436–4451. 10.7150/thno.54004 33754070 PMC7977448

[B63] Padovani C. Jevtić P. Rapé M. (2022). Quality Control of Protein Complex Composition. Mol. Cell 82, 1439–1450. 10.1016/j.molcel.2022.02.029 35316660

[B64] Palade G. E. Siekevitz P. Caro L. G. (1962). “Structure, Chemistry and Function of the Pancreatic Acinar Cell CIBA Fdn. Symposium,” in The Exocrine Pancreas, Normal and Abnormal Function. Editors de Reuck A. V. S. Cameron M.P.J. Cameron A.(United Kingdom: Churchill), 23–55.

[B74] Pavlin M. R Hurley J. H. (2020). The ESCRTs - Converging on Mechanism. J. Cell Sci. 133, jcs24333. 10.1242/jcs.240333 PMC7520454 32938689

[B65] Peng X. Li X. Yang S. Huang M. Wei S. Ma Y. Li Y. Wu B. Jin H. Li B. Tang S. Fan Q. Liu J. Yang L. Li H. (2021). LINC00511 Drives Invasive Behavior in Hepatocellular Carcinoma by Regulating Exosome Secretion and Invadopodia Formation. J. Exp. Clin. Cancer Res. 40, 183. 10.1186/s13046-021-01990-y 34088337 PMC8176717

[B66] Perissinotto F. Rondelli V. Senigagliesi B. Brocca P. Almásy L. Bottyán L. Merkel D. G. Amenitsch H. Sartori B. Pachler K. Mayr M. Gimona M. Rohde E. Casalis L. Parisse P. (2021). Structural Insights into Fusion Mechanisms of Small Extracellular Vesicles with Model Plasma Membranes. Nanoscale 13, 5224–5233. 10.1039/d0nr09075a 33687046

[B67] Pizzirani C. Ferrari D. Chiozzi P. Adinolfi E. Sandonà D. Savaglio E. Di Virgilio F. (2007). Stimulation of P2 Receptors Causes Release of IL-1β -loaded Microvesicles from Human Dendritic Cells. Blood 109, 3856–3864. 10.1182/blood-2005-06-031377 17192399

[B68] Puri N. Roche P. A. (2006). Ternary SNARE Complexes Are Enriched in Lipid Rafts during Mast Cell Exocytosis. Traffic 7, 1482–1494. 10.1111/j.1600-0854.2006.00490.x 16984405

[B69] Rabouille C. (2017). Pathways of Unconventional Protein Secretion. Trends Cell Biol. 27, 230–240. 10.1016/j.tcb.2016.11.007 27989656

[B70] Raffaele S. Lombardi M. Verderio C. Fumagalli M. (2020). TNF Production and Release from Microglia via Extracellular Vesicles: Impact on Brain Functions. Cells 9, 2145. 10.3390/cells9102145 PMC7598215 32977412

[B71] Ras-Carmona A. Gomez-Perosanz M. Reche P. A. (2021). Prediction of Unconventional Protein Secretion by Exosomes. BMC Bioinforma. 22, 333. 10.1186/s12859-021-04219-z PMC8210391 34134630

[B72] Raudenska M. Balvan J. Masarik M. (2021). Crosstalk between Autophagy Inhibitors and Endosome-Related Secretory Pathways: a Challenge for Autophagy-Based Treatment of Solid Cancers. Mol. Cancer 20, 140. 10.1186/s12943-021-01423-6 34706732 PMC8549397

[B73] Razzauti A. Laurent P. (2021). Ectocytosis Prevents Accumulation of Ciliary Cargo in *C. elegans* Sensory Neurons. Elife 10, e67670. 10.7554/eLife.67670 34533135 PMC8492061

[B75] Royo F. Théry C. Falcón-Pérez J. M. Nieuwland R. Witwer K. W. (2020). Methods for Separation and Characterization of Extracellular Vesicles: Results of a Worldwide Survey Performed by the ISEV Rigor and Standardization Subcommittee. Cells 9, 1955. 10.3390/cells9091955 PMC7563174 32854228

[B76] Rubartelli A. Bajetto A. Allavena G. Cozzolino F. Sitia R. (1993). Post-translational Regulation of Interleukin 1β Secretion. Cytokine 5, 117–124. 10.1016/1043-4666(93)90050-F 8334227

[B77] Rubartelli A. Cozzolino F. Talio M. Sitia R. (1990). A Novel Secretory Pathway for Interleukin-1 Beta, a Protein Lacking a Signal Sequence. EMBO J. 9, 1503–1510. 10.1002/j.1460-2075.1990.tb08268.x 2328723 PMC551842

[B78] Salvia R. Grimaldi A. Girardello R. Scieuzo C. Scala A. Bufo S. A. Vogel H. Falabella P. (2019). Aphidius Ervi Teratocytes Release Enolase and Fatty Acid Binding Protein through Exosomal Vesicles. Front. Physiol. 10, 715. 10.3389/fphys.2019.00715 31275155 PMC6593151

[B79] Schramm M. (1967). Secretion of Enzymes and Other Macromolecules. Annu. Rev. Biochem. 36, 307–320. 10.1146/annurev.bi.36.070167.001515 18257724

[B80] Shtam T. Evtushenko V. Samsonov R. Zabrodskaya Y. Kamyshinsky R. Zabegina L. Verlov N. Burdakov V. Garaeva L. Slyusarenko M. Nikiforova N. Konevega A. Malek A. (2020). Evaluation of Immune and Chemical Precipitation Methods for Plasma Exosome Isolation. PLoS One 15, e0242732. 10.1371/journal.pone.0242732 33232386 PMC7685508

[B81] Sitia R. Rubartelli A. (2020). Evolution, Role in Inflammation, and Redox Control of Leaderless Secretory Proteins. J. Biol. Chem. 295, 7799–7811. 10.1074/jbc.REV119.008907 32332096 PMC7261794

[B82] Somiya M. Kuroda S. i. (2021). Reporter Gene Assay for Membrane Fusion of Extracellular Vesicles. J. Extracell. Vesicles 10, e12171. 10.1002/jev2.12171 34807503 PMC8607979

[B83] Somiya M. (2020). Where Does the Cargo Go?: Solutions to Provide Experimental Support for the "extracellular Vesicle Cargo Transfer Hypothesis". J. Cell Commun. Signal. 14, 135–146. 10.1007/s12079-020-00552-9 32060725 PMC7272534

[B84] Song L. Chen J. Sun A. Schekman R. (2021). APEX-mediated Proximity Labeling of Proteins in Cells Targeted by Extracellular Vesicles. Bio-protocol 11, e4213. 10.21769/BioProtoc.4213 34859128 PMC8595426

[B85] Sun M. Xue X. Li L. Xu D. Li S. Li S. C. Su Q. (2021). Ectosome Biogenesis and Release Processes Observed by Using Live-Cell Dynamic Imaging in Mammalian Glial Cells. Quant. Imaging Med. Surg. 11, 4604–4616. 10.21037/qims-20-1015 34737927 PMC8511719

[B86] Tseng C.-C. Dean S. Davies B. A. Azmi I. F. Pashkova N. Payne J. A. Staffenhagen J. West M. Piper R. C. Odorizzi G. Katzmann D. J. (2021). Bro1 Stimulates Vps4 to Promote Intralumenal Vesicle Formation during Multivesicular Body Biogenesis. J. Cell Biol. 220, e202102070. 10.1083/jcb.202102070 34160559 PMC8240856

[B87] Van Niel G. D’Angelo G. Raposo G. (2018). Shedding Light on the Cell Biology of Extracellular Vesicles. Nat. Rev. Mol. Cell Biol. 19, 213–228. 10.1038/nrm.2017.125 29339798

[B88] Verweij F. J. Bebelman M. P. Jimenez C. R. Garcia-Vallejo J. J. Janssen H. Neefjes J. Knol J. C. de Goeij-de Haas R. Piersma S. R. Baglio S. R. Verhage M. Middeldorp J. M. Zomer A. van Rheenen J. Coppolino M. G. Hurbain I. Raposo G. Smit M. J. Toonen R. F. G. van Niel G. Pegtel D. M. (2018). Quantifying Exosome Secretion from Single Cells Reveals a Modulatory Role for GPCR Signaling. J. Cell Biol. 217, 1129–1142. 10.1083/jcb.201703206 29339438 PMC5839777

[B89] Walter P. Blobel G. (1981). Translocation of Proteins across the Endoplasmic Reticulum. II. Signal Recognition Protein (SRP) Mediates the Selective Binding to Microsomal Membranes of In-Vitro-Assembled Polysomes Synthesizing Secretory Protein. J. Cell Biol. 91, 551–556. 10.1083/jcb.91.2.551 7309796 PMC2111991

[B90] Walter P. Gilmore R. Blobel G. (1984). Protein Translocation across the Endoplasmic Reticulum. Cell 38, 5–8. 10.1016/0092-8674(84)90520-8 6088076

[B91] Wang J. Zhuang X. Greene K. S. Si H. Antonyak M. A. Druso J. E. Wilson K. F. Cerione R. A. Feng Q. Wang H. (2021). Cdc42 Functions as a Regulatory Node for Tumour‐derived Microvesicle Biogenesis. J. Extracell. Vesicles 10, e12051. 10.1002/jev2.12051 33473262 PMC7804048

[B92] Yang J.-M. Gould S. J. (2013). The Cis-Acting Signals that Target Proteins to Exosomes and Microvesicles. Biochem. Soc. Trans. 41, 277–282. 10.1042/BST20120275 23356297

[B93] Yang L. Peng X. Li Y. Zhang X. Ma Y. Wu C. Fan Q. Wei S. Li H. Liu J. (2019). Long Non-coding RNA HOTAIR Promotes Exosome Secretion by Regulating RAB35 and SNAP23 in Hepatocellular Carcinoma. Mol. Cancer 18, 78. 10.1186/s12943-019-0990-6 30943982 PMC6446409

[B94] Yang Y. Qin M. Bao P. Xu W. Xu J. (2017). Secretory Carrier Membrane Protein 5 Is an Autophagy Inhibitor that Promotes the Secretion of α-Synuclein via Exosome. PLoS One 12, e0180892. 10.1371/journal.pone.0180892 28700687 PMC5507457

[B95] Zhang M. Liu L. Lin X. Wang Y. Li Y. Guo Q. Li S. Sun Y. Tao X. Zhang D. Lv X. Zheng L. Ge L. (2020). A Translocation Pathway for Vesicle-Mediated Unconventional Protein Secretion. Cell 181, 637–652.e15. 10.1016/j.cell.2020.03.031 32272059

[B96] Zhao X. Guan Y. Liu F. Yan S. Wang Y. Hu M. Li Y. Li R. Zhang C. X. (2022). SNARE Proteins Mediate α-Synuclein Secretion via Multiple Vesicular Pathways. Mol. Neurobiol. 59, 405–419. 10.1007/s12035-021-02599-0 34705229

[B97] Zhao Y. G. Zhang H. (2019). Autophagosome Maturation: An Epic Journey from the ER to Lysosomes. J. Cell Biol. 218, 757–770. 10.1083/jcb.201810099 30578282 PMC6400552

[B98] Zhao Z. Kesti T. Uğurlu H. Baur A. S. Fagerlund R. Saksela K. (2019). Tyrosine Phosphorylation Directs TACE into Extracellular Vesicles via Unconventional Secretion. Traffic 20, 202–212. 10.1111/tra.12630 30569492

[B99] Zhu Q. Yamakuchi M. Lowenstein C. J. (2015). SNAP23 regulates endothelial exocytosis of von Willebrand factor. PLoS One 10, e0118737. 10.1371/journal.pone.0118737 26266817 PMC4534191

